# Nissen fundoplication and combined procedures to reduce recurrence of gastroesophageal reflux disease in neurologically impaired children

**DOI:** 10.1038/s41598-020-68595-x

**Published:** 2020-07-15

**Authors:** Emanuela Ceriati, P. Marchetti, R. Caccamo, O. Adorisio, F. Rivosecchi, F. De Peppo

**Affiliations:** 1 Department of Surgery, “Bambino Gesù” Research Children’s Hospital, Via della Torre di Palidoro, snc-Passoscuro, Rome, Italy; 2 Via Barbarano Romano, 7, 00189 Rome, Italy

**Keywords:** Health sciences, Paediatric research, Malnutrition, Gastro-oesophageal reflux disease

## Abstract

Neurologically impaired children account for almost half of the fundoplication procedures performed for gastroesophageal reflux disease. Aim of the present study was to report results of antireflux surgery in neurologically impaired children. A retrospective study of neurologically impaired children who underwent fundoplication over a 13-year period (1999–2012) was performed. Recurrence rate, major complications and parents/caregivers perceptions of their child's quality of life following antireflux surgery were analyzed. A total of 122 children (median age: 8 years 9 months; range: 3 months to 18 years) had open “tension-free” Nissen fundoplication, gastrostomy + /− pyloroplasty. Gastroesophageal reflux disease was in all cases documented by at least two diagnostic exams. Median duration of follow-up was 9.7 (1.9–13) years. Three (2.4%) recurrences were documented and required surgery re-do. Major complications were 6%. Seventy-nine of 87 (90%) caregivers reported that weight gain was improved after fundoplication with a median score of 1 (IQR: 1–2). Significant improvement was perceived in postoperative overall quality of life. In this series of fundoplication recurrence incidence was low, serious complications were uncommon and caregivers’ satisfaction with surgery was high. Accurate patient’s selection and creating a “low-pressure” surgical system are mandatory to obtain these results.

## Introduction

It is well known that children with neurological impairment (NI) develop gastroesophageal reflux disease (GERD) more frequently (26–91%) than normal individuals (5–7%)^1–3^.

NI children constitute a great part of the population of patients who require surgery for GERD. Nissen FP is the standard surgical therapy for GERD. Keeping in mind that reported rates of failure depend on definitions used and duration of follow-up, large contemporary series of FP report a requirement for re-do surgery in 15–30%^4–7^.

This higher mortality, morbidity, surgical complication and re-operation rate leads to a reluctance on the part of some surgeons to undertake this procedure. The burden of GERD related problems is known to have significant psychosocial implications and adversely impacts on the quality of life of these children and their caregivers but there is a limited understanding of their impact on well-being and quality of life.

Thus the present work does not aim only at analyzing retrospectively yet another series of FP to add to the several large ones already published^8–12^ but focus on:

- Selection and preoperative criteria for tight indications to surgical treatment;
- Technical suggestions giving excellent results in term of complications and failure rate;
- Parent/caregiver perceptions of symptom following surgery, with evaluation of their attitude to surgical intervention.

## Methods

Our Institution is a tertiary care referral centre for treatment of children affected by NI. All patients were treated with drug therapy and the indication for surgery was persistence of clinical signs under medication or drug-dependence. GERD was in all cases suspected on the basis of clinical history and invariably documented by at least two of the following exam: Gastrointestinal (UGI) series, 24-pH-metry study, Esophagogastroduodenoscopy (EGD) and Scintigraphy of the oesophageal-gastric tract (radio nuclide-labeled 99mTc sulfur colloid liquid or semisolid food). Gastric emptying was defined as *delayed* in children younger than 5 years when more than 65% intragastric retention was observed at 60 min after radiolabeled meal completion and when more than 50% retention was observed in children older than 5 years^13^. A “tension-free” FP was performed with a xifo**-**umbilical approach according to Nissen technique^14^.

Technical details:

1. Nissen wrap secured with 2–3 interrupted non-absorbable sutures (lowest stitch anchored to oesophageal wall)
2. No stitches to anchor the wrap to diaphragmatic crura
3. Extended division of the short gastric vessels performed when necessary to obtain a “loose” wrap especially in the presence of gastric fundus hypoplasia.
4. Heineke-Mickulicz pyloroplasty (absorbable sutures and occasionally an overlaid omental patch to protect against leakage) in all patients with documented DGE
5. Temporary or permanent Stamm gastrostomy^15^.

All these surgical steps aimed to obtain a “low-pressure” surgical system.

Clinical evaluation (vomiting, retching, recurrent respiratory tract infections) was performed 2 months after surgery and at least once/year. In case of “alarm” symptoms (anaemia, persisting retching/vomiting) or in a range time of 12 to 36 months, an instrumental follow-up (trans-gastrostomy endoscopy or barium meal) was performed. To evaluate parents/caregiver perceptions of their child's quality of life following antireflux surgery we adapted the questionnaire designed by O’Neill et al.^23^ that evaluated perceptions of child well-being and care requirements, parental/caregiver attitude and overall satisfaction with the surgical results. Parents/caregivers were invited to fill in the questionnaire 2 years before and 2 years after surgery and they were asked to complete it when they attended for clinical review or by telephone (contacted by the same clinician, EC)”.

Our study was approved by the Ethics Committee of Bambino Gesù Hospital, in compliance with the Helsinki Declaration and was performed in accordance with relevant guidelines and regulations. Informed consent was obtained from parents of children enrolled in our study.

### Data analysis

Information collected retrospectively was stored in a Microsoft Excel database. Data are quoted as median or percentage of total unless otherwise indicated. Parametric data were analysed with the Student’s t test. P values of less than 0.05 were considered statistically significant.

## Results

The medical records of NI children who underwent Nissen FP from 1999 to 2012 were reviewed after Institutional Review Board approval. Data collected included: demographics, neurological status, indications to surgery, pre-operative work-up, concomitant procedures, intraoperative and postoperative mortality and complication rates.

From January 1999 through December 2012, 122 Nissen FP with an open approach in NI children have been performed in our Institution. In all cases (if no already performed) a Stamm gastrostomy procedure was associated. If a delayed gastric emptying was documented a Heineke-Mickulicz pyloroplasty was also performed. There were 58 boys and 64 girls; the median age was 8 years 9 months (range = 3 months to 18 years). Only two patients younger than 12 months were operated on because of recurrent vomiting associated to technical difficulties to perform a percutaneous endoscopic jejunostomy. Table 1 illustrates causes of neurological impairment. Indication for surgery was always a symptomatic GERD presented by upper gastrointestinal symptoms, (vomiting, regurgitation, dysphagia, swallowing difficulties, hematemesis and anemia) and extra gastrointestinal symptoms (aspiration pneumonia, recurrent bronchopneumonia, recurrent respiratory tract infections, failure to thrive). This was respectively 65% (n = 79) and 35% (n = 43)—Table 2. Gastroesophageal reflux disease was in all cases diagnosed on the basis of clinical history and invariably documented by at least two of the following exam: barium meal, 24-pH-study, endoscopy and scintigraphy of the oesophageal-gastric tract (radio nuclide-labeled 99mTc sulfur colloid liquid or semisolid food) as shown in Table 3. The latter was performed in all children but the first 16 of this series (equipment not available in our Hospital). All the surgical procedures were performed by the same surgeon (FDP) and team.

**Table 1:** Causes of neurological impairment.

	n	%
Perinatal anoxia injury/asphyxia	57	45
Chromosomal abnormalities	27	22
CNS anomalies	17	13
CNS infections	9	7
Metabolic disease	6	5
Postnatal hypoxic ischemic brain injury	3	2
Postvaccinal encephalitis	2	1
CNS tumour	1	1

*CNS* central nervous system.

**Table 2:** GERD symptoms.

	n	%
**Upper GI symptoms**	79	64
Vomiting/regurgitation/dysphagia	62	78
Hematemesis	11	14
Anaemia	6	7
**Extra GI symptoms**	43	35
Aspiration pneumonia	27	63
Recurrent broncopneumonia/respiratory tract infections	16	37

**Table 3:** Technical investigations.

	n	%
Endoscopy + scintigraphy	58	47
Endoscopy + scintigraphy + 24-h pH study	33	27
24-h pH study + endoscopy	15	12
24-h pH study + scintigraphy	5	4
Endoscopy + UGI	4	3
24-h pH study + UGI	3	2
UGI + endoscopy + scintigraphy	2	1
Scintigraphy + 24-h pH study + UGI	1	1
Endoscopy + scintigraphy + 24-h pH study + UGI	1	1

Forty-eight (40%) patients underwent to a Nissen with associated gastrostomy and pyloroplasty while in 24 (20%) patients a Nissen with gastrostomy was performed. In 32 (64%) of the 50 gastrostomy-carriers patients at time of surgery a re-do Stamm gastrostomy was necessary to difficult mobilization of gastric fundus for gastrostomy’s tension. A Nissen with associated pyloroplasty was performed in 23 (19%) cases; while only Nissen in 27 (22%) of them (gastrostomy-carriers). In total, 71 (58%) patients underwent to Heineke-Mickulicz pyloroplasty.

No intra-operative mortality has been reported. All patients with no dysphagia or swallowing difficulties were discharged with a soft /bland oral diet for the first 2–4 weeks; unrestricted feedings (if no documented alimentary allergy) were allowed since then. In patients with malnutrition an enteral implementation on a continuous basis during nighttime with a nutritionally complete normo or hypercaloric formula was recommended. In patients with documented dysphagia enteral nutrition was administered usually by bolus during daytime and on a continuous basis during nighttime. Median duration of follow-up was 9.7 (1.9–13) years. To date, eight children (6%) have died postoperatively: one child died 20 days postoperatively for complications of pneumonia. Seven patients died, more than 2 years after the operation because of their progressive neurological disease. Three patients (2.4%) in the study group experienced episodes of vomiting, as well as recurrent respiratory tract infections due to a recurrent GERD documented by UGI (through gastrostomy) respectively after 3, 6 and 18 months after surgery. In all of them a redo Nissen FP was performed. Major complications requiring surgery occurred in six patients (5%). The complications consisted of haemorrhage in four cases (3%); intestinal occlusion in 2 (1.5%). The questionnaire response rate was 75.4% (87/116 parents/caregivers contacted). Seventy-nine of 87 (90%) caregivers reported that weight gain was improved after FP with a median score of 1 (IQR: 1–2). With regard to daily care and overall condition of the child (Table 4) significant improvement was perceived in the ease of feeding, child’s comfort, comfort during feeding and child’s ability to enjoy life.

**Table 4:** Parent/caregiver attitudes regarding daily care and the overall condition of their child.

1	**Ease of feeding**	
	2 years before surg In the last 2 years	4.78 ± 0.74 2.59 ± 0.79*
2	**Comfort during feeding**	
	2 years before surg In the last 2 years	4.15 ± 1.07 2.79 ± 0.80*
3	**Child’s comfort**	
	2 years before surg In the last 2 years	3.76 ± 0.87 2.69 ± 0.69*
4	**Child’s ability to enjoy life**	
	2 yrs before surg In the last 2 yrs	3.75 ± 0.57 3.01 ± 0.73*
5	**Child’s developmental progress**	
	2 years before surg In the last 2 years	4.00 ± 0.74 3.62 ± 0.62

*Score derived from a 5-point Likert scale (1: excellent; 2: good: 3: average; 4: poor; 5: terrible).

*p < .05 versus attitude 2 years before surgery.

With respect to parent/caregiver attitudes regarding their child’s and their own quality of life (Table 5) caring for the child was much easier postoperatively with a significant improvement of postoperative overall quality of life. Eighty-four families (96%) stated that the operation turned out better than or about as they had expected.

**Table 5:** Child and parents/caregiver quality of life (6–8).

1	**Overall ease of caring for your child**	
	2 years before surg In the last 2 years	3.63 ± 0.83 2.18 ± 0.58*
2	**Quality of time spent with your child**	
	2 years before surg In the last 2 years	2.45 ± 0.77 2.14 ± 0.35
3	**Your overall quality of life**	
	2 years before surg In the last 2 years	3.55 ± 0.79 2.17 ± 0.48*

*Score derived from a 5-point Likert scale (1: excellent; 2: good: 3: average; 4: poor; 5: terrible).

*p < .05 versus attitude 2 years before surgery.

## Discussion

The management of GERD in NI children is controversial, and the outcomes of different procedures adopted are difficult to analyse. When a child with cerebral palsy is referred for surgical treatment of GERD, it seems reasonable to expect a safe result, aiming to a permanent cure of reflux. Unfortunately, making reference to the published results on this topic we are not able to guarantee these outcomes to patients’ families, so far. Most paediatric surgeons when have to decide about the need for surgery do not perform preoperative investigations other than a review of the clinical history and physical examination, taking decisions based only on symptomatic assessment and failure to respond to medical therapy^18^. Moreover, there is no gold standard technique to diagnose GERD in children with NI. The North American Society of Pediatric Gastroenterology, Hepatology, and Nutrition (NASPGHAN) guidelines for GERD are unclear for such patients^19^. Candidates for surgery should undergo appropriate diagnostic investigation to ensure that GERD is chronic and relapsing and it is the correct final diagnosis. We generally consider the entire clinical scenario in combination with the studies available. In patients with severe NI, different mechanisms combine to determine the gastriç emptying process depending on the balance between propulsive and resistance forces to outflow, such as gastric accommodation and tone, antral contractivity, pyloric function, antroduodenal coordination and entero-enteric refluxes. The association of GERD and gastric emptying in children is controversial and gastric motility following FP remains poorly understood. In our experience, if gastric scintigraphy presents a delayed gastric emptying, we advocate pyloroplasty at the same time as FP, to improve gastric emptying. This procedure lessens outflow resistance promoting gastric emptying. In this series pyloroplasty extended of about 10–15 min the length of surgery but didn't affect morbidity or mortality rate. Aim of surgical treatment is to create a mechanical antireflux barrier by a valve/ high-pressure zone mechanism at the distal oesophagus, through the re-placement of the distal oesophagus into the abdominal cavity. Creating a high-pressure zone can cause high-tension facilitating wrap's slipping or disruption. The combination of “tension-free” Nissen procedure, gastrostomy (100%) and possible pyloroplasty (58%) is what we define as “low-pressure” procedure acting as a “control pressure device as it creates a sort of “pressure-valve” aimed to reduce gastric outflow resistance in case, for instance, of retching episodes. That’s why we always suggest and teach parents and caregivers especially in the early postoperative period to open the gastrostomy and to check gastric content before any bolus feeding. The target is to obtain a lower outflow resistance and to reduce risk of a high-tension wrap’s slipping or disruption causing a relapsing GERD.

In our experience the gastrostomy performed at the same time of FP, even in NI patients with no severe documented dysphagia, aimed to improve the nutritional status and feeding management of early and late-postoperative course and our results in terms of complications, morbidity and especially failure rate are very comfortable, so far. Comparison of traditional open surgery and laparoscopic surgery is beyond the scope of this manuscript. The most effective way of ensuring the success of the surgical procedure is to create a wrap over the oesophagus just in proximity of the gastroesophageal junction, below the diaphragm, and to keep it in that position on a permanent basis. This can be reached both by an open or a laparoscopic approach. A recent systematic review by the American Pediatric Surgical Association Outcomes and Evidence-Based Practice Committee concluded that laparoscopic FP may be comparable to open FP; data on long-term effectiveness are missing and do not provide sufficient evidence to formulate recommendations^20^. In NI children gastrojejunal (GJ) feeding is increasingly regarded as a popular alternative to surgery to treat GERD. Long-term GJ feeding has been proposed by some as an effective option to treat reflux particularly in NI patients^21,22^. We adopt this technique in case of patients younger than 1 year with failure to thrive (in our series only 2 patients under 2 years of age were operated on) to let them grow up and, if a GERD is confirmed, to perform surgery later on, or if parents refuse surgery. However, it commits to continuous pump feeding to avoid dumping, and tube blockage/dislodgement are frequently such to complicate this technique. There is also evidence of duodenogastric reflux of bile in many patients with GJ feeding. To choose a questionnaire to assess GERD-related symptoms was challenging, given the lack of validated carer-reported outcome measures in this population. Very few studies have been published on caregiver perceptions of antireflux procedures in NI children^16,23,24^. To be considered successful, surgery for GERD treatment in NI patients must improve quality of life not only for the child but also for the parents and/or caregivers. In the present series the satisfaction of parents/caregivers with regard to this operation is documented, not only as far as the ease of care is concerned, but also regarding the quality of time spent with the children. We are aware of the fact that our study has several limitations (retrospective study, personal series, no control group, risk of bias in reporting), but since indications for and outcomes after FP in NI children are still under discussion, our aim was to present a NI group who underwent surgical treatment of GERD, by one of the available access (laparotomy), with good results in terms of recurrence and complications rate. Dealing with this group of patients cast some ethical points that have always to be considered and that make difficult to have rigorous and scientifically high-quality paper. Primary target outcomes after treatment of GERD in NI children are the cure of the reflux and the improvement of quality of life. Prospective studies are needed to determine which is the preferred treatment of GERD in these patients. From our point of view, aim of our treatment is to heal children from GERD and to improve their quality of life. Improving quality of life of caregivers who take care of these patients means improving quality of life of children as well and if FP, in selected patients and with high success rate makes easier to do this, then we believe it is right to perform it.
